# The Elusive Antifibrotic Macrophage

**DOI:** 10.3389/fmed.2015.00081

**Published:** 2015-11-13

**Authors:** Adhyatmika Adhyatmika, Kurnia S. S. Putri, Leonie Beljaars, Barbro N. Melgert

**Affiliations:** ^1^Department of Pharmacokinetics, Toxicology and Targeting, Groningen Research Institute for Pharmacy (GRIP), University of Groningen, Groningen, Netherlands; ^2^Department of Pharmaceutical Technology and Biopharmacy, Groningen Research Institute for Pharmacy (GRIP), University of Groningen, Groningen, Netherlands; ^3^Faculty of Pharmacy, University of Indonesia, Depok, Indonesia; ^4^GRIAC Research Institute, University Medical Center Groningen, University of Groningen, Groningen, Netherlands

**Keywords:** macrophages, antifibrotic, fibrosis, resolution, monocytes, MMP, cathepsin K, polarization

## Abstract

Fibrotic diseases, especially of the liver, the cardiovascular system, the kidneys, and the lungs, account for approximately 45% of deaths in Western societies. Fibrosis is a serious complication associated with aging and/or chronic inflammation or injury and cannot be treated effectively yet. It is characterized by excessive deposition of extracellular matrix (ECM) proteins by myofibroblasts and impaired degradation by macrophages. This ultimately destroys the normal structure of an organ, which leads to loss of function. Most efforts to develop drugs have focused on inhibiting ECM production by myofibroblasts and have not yielded many effective drugs yet. Another option is to stimulate the cells that are responsible for degradation and uptake of excess ECM, i.e., antifibrotic macrophages. However, macrophages are plastic cells that have many faces in fibrosis, including profibrotic behavior-stimulating ECM production. This can be dependent on their origin, as the different organs have tissue-resident macrophages with different origins and a various influx of incoming monocytes in steady-state conditions and during fibrosis. To be able to pharmacologically stimulate the right kind of behavior in fibrosis, a thorough characterization of antifibrotic macrophages is necessary, as well as an understanding of the signals they need to degrade ECM. In this review, we will summarize the current state of the art regarding the antifibrotic macrophage phenotype and the signals that stimulate its behavior.

## Introduction

Fibrosis is a serious complication associated with aging and with chronic injury and inflammation within an organ. It is characterized by progressive and irreversible destruction of normal architecture of an organ by excessive deposition of extracellular matrix (ECM). The excess ECM ultimately leads to organ malfunction and death because there are no effective therapies to stop or reverse fibrosis development. A mechanistic understanding of how ECM homeostasis is maintained in healthy situations, the similarities and differences between the various organs, and how it becomes dysregulated in fibrosis is of vital importance for defining novel targets for therapy. More insight into these processes will help the development of novel antifibrotic drugs.

Production of ECM is part of a normal repair response after tissue damage. Tissue repair has distinct stages, including a clotting phase, an inflammatory phase, a (myo)fibroblast proliferation phase, and a remodeling phase in which normal tissue architecture is restored (1). During the remodeling phase, myofibroblasts produce ECM and promote tissue contraction, which will ultimately lead to resolution of the damage. The current dogma is that ongoing microinjury within an organ induces an imbalance in ECM homeostasis and subsequently leads to fibrosis (2, 3). In most organs, ECM-producing myofibroblasts are found in close proximity with macrophages, and there is increasing evidence that suggests that normally these two cell types interact in many ways to control ECM homeostasis and that these interactions may be dysregulated in fibrosis (3–6). Myofibroblasts, as the major producers of ECM, have been the focus of fibrosis research for many years. Unfortunately, this has not yielded many successful drugs yet. Therefore, the role macrophages have in controlling ECM production in fibrosis has been getting more attention recently.

Macrophages are important cells in all stages of the fibrotic process (7). On the one hand, they have been found to promote fibrosis by secreting profibrotic mediators such as transforming growth factor beta (TGFβ) and platelet-derived growth factor (PDGF) that induce proliferation and activation of myofibroblast (7–9). On the other hand, they also facilitate the resolution of fibrosis by producing specific matrix metalloproteinases (MMPs) and other proteolytic enzymes like cathepsins that degrade fibrotic ECM, and they express receptors that can phagocytose pieces of degraded ECM (10). Studies in models of pulmonary and liver fibrosis have shown that when macrophages are depleted during the early, inflammatory phase of fibrosis, ECM deposition was reduced but when they are depleted during the remodeling phase, ECM deposition was aggravated (8–11). These studies elegantly showed that the behavior of macrophages is highly plastic, but it remains unclear how the pro- and antifibrotic activities of macrophages are regulated. Knowing which signals induce antifibrotic behavior of macrophages is particularly important because restoration of normal tissue architecture can only proceed if the deposited excess ECM is removed. These signals may subsequently be used for the development of a whole new class of antifibrotic drugs. However, discerning antifibrotic macrophages from other macrophages is difficult, since characteristic markers are unclear, as are the signals that induce antifibrotic macrophages.

In this review, we will discuss evidence currently present in the literature that enables us to identify antifibrotic macrophages and the signals that are needed to induce them in order to design macrophage-directed antifibrotic therapeutics. Studies used for this review were gathered by a systematic search of Pubmed using the keywords “macrophages,” “fibrosis,” and “(resolution OR antifibrotic).” Only studies discussing pro- or antifibrotic activities of macrophages or phenotypical markers of these macrophages were included.

## Macrophage Plasticity

Macrophages have many roles in the immune system and are strongly involved in fighting microbial threats, inflammation, repair and resolution to return to homeostasis. For years, researchers have tried to define distinct macrophage polarization states or phenotypes that are responsible for these different tasks (12). They have been classified in several different ways, mostly into two main groups with M1 macrophages as the classically activated macrophages and M2 macrophages as the alternatively activated macrophages (13, 14). Broadly speaking, M1-activated macrophages are associated with inflammatory responses and are involved in fighting infections. This phenotype develops after exposure to microbial products, and proinflammatory cytokines such as tumor necrosis factor alpha (TNFα) and interferon gamma (IFNγ). M2-activated macrophages are more difficult to capture into one phenotype, and this has led to the suggestion to group them into the different subsets M2a, M2b, and M2c (15). These subsets are associated with repair processes and resolution of inflammation and are induced by a variety of signals such as interleukin-4/interleukin-13 (IL-4/IL-13) for M2a, immune complexes and lipopolysaccharides (LPS) for M2b, and IL-10/TGFβ/glucocorticosteroids for M2c. This classification had its uses for well-controlled *in vitro* experiments but could not capture the multitude or spectrum of polarization states present *in vivo* leading to much confusion in the field. This has led to the suggestion to identify macrophages through their origin, the polarizing substance, and/or markers they do or do not express (16).

The confusion about macrophage polarization is also apparent in the field of fibrosis. The widespread use of the M1/M2 classification has lead to the suggestion that M1 macrophages promote inflammation in the inflammatory stages of wound repair and subsequently polarize to or are being replaced by M2 macrophages that promote fibrosis. However, the complex microenvironment macrophages are exposed to *in vivo* has many stimuli that induce different functions that cannot be captured in M1 and M2. Furthermore, the M2 phenotype is a complex collection of divergent activities that are sometimes even contradictory. For example, in mice, M2 macrophages have been described by their expression of arginase-1 (Arg-1), and these macrophages were considered to be profibrotic. However, Pesce et al. showed, using macrophage-specific Arg-1-knockout mice, that these Arg-1-expressing macrophages were actually responsible for suppressing fibrosis development (17). This intriguing result shows the plasticity of profibrotic and antifibrotic behaviors within the M2 macrophage subset in a complex tissue environment.

Other studies have circumvented the M1/M2 dichotomy by naming macrophages after their roles in inflammation and tissue remodeling: i.e., proinflammatory, profibrotic, proresolution, resolving, or scar-associated macrophages (4, 10, 18–20). For the purpose of this review, we will be specifically addressing the macrophages that are associated with areas of existing fibrosis and are responsible for clearing away excess ECM, also known as proresolution or antifibrotic macrophages.

## Murine Versus Human Macrophages

The discovery of macrophages phenotypes has largely been driven by murine models. Translation to human steady-state conditions or diseases is scarce and hampered by the fact that many phenotypical and functional markers are murine-specific, and the human counterparts are unknown (12, 21). For instance, the widely used M2 markers Ym1 (chitinase 3-like protein 3) and FIZZ1 (resistin-like molecule alpha 1/found in inflammatory zone 1) are only expressed on murine IL-4/IL-13-activated macrophages and not in their human counterparts. Though firmly associated with development of fibrosis in mouse models, how these markers themselves play a role is unclear (22–24), making it even more difficult find their human equivalents. Most of the information on antifibrotic macrophages will therefore be derived from murine studies. Whenever possible we will try to make the translation to the human situation.

## The Origin of Tissue Macrophages

Mature macrophages in adult tissues can originate from two different sources: either from circulating blood monocytes that infiltrate the tissues after birth or from embryonic macrophages infiltrating tissues before birth and that self-maintain throughout life (25–32). The distinction between hematopoetic versus embryonic origin may be important because this may determine their functionality (33). For instance, liver-resident alternatively activated macrophages were found to be phenotypically and functionally distinct from monocyte-derived alternatively activated macrophages. The first were found to be key in suppressing schistosomiasis-induced chronic inflammation, while the latter monocyte-derived ones could slow the progression of fibrosis (34).

Recent experiments have shown that during steady-state conditions, in most organs, tissue macrophages are of embryonic origin (25–32). These embryonic macrophages can develop from yolk sac macrophages directly or, through erythro-myeloid progenitors in the fetal liver (25, 30, 35, 36). In the developing embryo, hematopoiesis begins in the yolk sac with primitive erythrocytes and macrophages developing in the absence of hematopoietic stem cells and spreading into developing peripheral tissues (37). This primitive hematopoiesis is not sufficient to support the developing embryo until hematopoietic stem cells are functional. Therefore, a second wave of hematopoiesis is supported by erythro-myeloid progenitors migrating from the yolk sac to the fetal liver until the hematopoietic stem cells are ready to take over after birth (36). During this period of primitive hematopoiesis, macrophages spread via the blood into peripheral tissues of the fetus, giving rise to tissue-resident macrophages that self-maintain throughout life (38). Several organs including spleen, pancreas, and kidney exhibit mixed contribution from embryonic and hemopoietic stem cell-derived procursors (38). Like other tissue macrophages, intestinal macrophages are also first established before birth from embryonic precursors. However, unlike macrophages in most other tissues, these embryonic macrophages in the gut are replaced shortly after birth by blood monocyte-derived macrophages. Thus, intestinal macrophages appear to be entirely derived from circulating monocytes (39, 40). An overview of the origins of macrophages in the different tissues can be found in Table 1.

**Table 1:** **An overview of the origins of macrophages in the different tissues**.

Tissue-resident macrophages	Embryonic progenitor		Adult hematopoietic stem cells
	Yolk sac	Fetal liver monocytes	
Spleen (27)		√	√
Pancreas (27)		√	√
Kidney(27)		√	√
Brain (microglia) (41)	√		
Heart (31)	√	√	√ (small number)
Skin (Langerhans cells) (30)		√	
Skin (dermal macrophages) (42, 43)			√
Gut (39, 40)			√
Lung (alveolar macrophages) (25)		√	
Liver (Kupffer cell) (28, 44)	√	√	

Resident tissue macrophages normally have homeostatic functions including clearing up debris and apoptotic cells, first-line defense against microbial threats, downregulating unnecessary inflammatory responses of the tissue, and contribution to normal ECM turnover. In cases of tissue damage, the steady-state conditions change, and the tissue-resident macrophages may be supplemented with macrophages derived from incoming monocytes to fight incoming threats and help wound healing. In mice, two populations of monocytes have been identified based on the expression of the surface molecule lymphocyte antigen 6C (Ly6C). Monocytes with high expression of Ly6C are generally called classical or inflammatory monocytes, and these patrol the extravascular tissues in homeostatic conditions (29). During this patrolling function, they remain monocytic and do not commit to being macrophages. During inflammation, however, they respond with rapid extravasion into the affected tissues and they can readily transform into macrophages with limited potential for migration (29). Monocytes with low expression of Ly6C are called non-classical monocytes and patrol the blood vessels to monitor endothelial cell homeostasis (45, 46). They develop from the Ly6C-hi subset (26, 47, 48), and this can also take place in injured or inflamed tissue with subsequent conversion to wound-healing macrophages that can proliferate locally (49, 50).

In humans, similar monocytes’ subsets are found based on the expression of CD14 and CD16 (51). Classical monocytes express high levels of CD14 and no CD16, while non-classical monocytes express high levels of CD16 and low levels of CD14. Both in humans and mice, an intermediate third subset is suggested to exist characterized in humans by high levels of CD14 and intermediate levels of CD16. The functions of this subset are not well understood, although they have been found to preferentially accumulate in inflamed human livers and have been postulated to play a role in fibrogenesis (52).

Unfortunately, there are no reliable markers to distinguish between macrophages from embryonic or hematopoietic/monocytic origin, which makes it difficult to study the contributions of the two types of macrophages to changes in homeostatic conditions, especially in humans. In mice, some lineage-tracing studies have been performed with special mouse models in the context of fibrosis to get some insight into the origin of macrophages in fibrotic tissues, and these studies are discussed below.

## The Origin of Macrophages During Fibrosis

Several papers have investigated the various origins of macrophages in the context of fibrosis. There is a clear role for infiltrating Ly6C-hi monocytes in fibrosis. These monocytes have high expression of CCR2 (C–C motif chemokine receptor type 2) and have been shown to CCR2-dependently infiltrate the kidney, liver, heart, and lung after acute injury (8, 53–56). Less fibrosis is found when this migration is prevented either by specific depletion of the Ly6C-hi subset or by interfering with CCR2 function (53, 57). In liver and lung, it was shown that Ly6C-hi monocytes clearly facilitate the progression of fibrosis, but without obviously engrafting into the tissue as macrophages, which may indicate that their patrolling behavior of extravascular tissues is not restricted to steady-state conditions (8, 53).

Many models of fibrosis consist of toxic injury (e.g., carbon tetrachloride and bleomycin) with an acute inflammatory phase followed by a fibrotic phase and a resolution phase with a return to fairly normal tissue structure. In these models, it was shown that depletion of macrophages in the resolution phase slowed down the process of resolution (8, 18, 57–62). These restorative macrophages appear to be derived from the recruited Ly6C-hi monocytes that undergo a phenotypic switch to a Ly6C-lo phenotype (18, 57). However, in a study by Baeck et al. inhibiting a transient CCR2-dependent accumulation of Ly6C-hi monocytes in the resolution phase accelerated scar resolution in two models of hepatic fibrosis (62). Therefore, contributions of both recruited Ly6C-lo monocytes and tissue-resident macrophages are also likely (8, 59–61). Corroboration for involvement of Ly6C-lo monocytes comes from a study showing that deletion of the fractalkine receptor CX3CR1 (C–X3–C motif chemokine receptor 1), which is highly expressed on Ly6C-lo monocytes, inhibits resolution of hepatic fibrosis (60). Gibbons et al. showed that ablation of tissue-resident macrophages in the lung during the resolution phase of bleomycin-induced injury also slowed down resolution (8).

In conclusion, macrophages of various origins, hematopoietic and embryonic, contribute to fibrosis and its resolution. The evidence available points at antifibrotic macrophages being either derived from CX3CR1-expressing Ly6C-lo monocytes and/or embryonically derived tissue-resident macrophages, while ly6C-hi monocytes appear to be profibrotic. For a summary of the available data also see Table 2.

**Table 2:** **Origins of antifibrotic macrophages**.

Organ	Antifibrotic macrophages	
	Tissue resident	Ly6C-lo-recruited monocyte
Peritoneal	√ (63)	√ (63)
Lung	√ (8, 64, 65)	√ (59, 66)
Liver (Kupffer cell)		√ (60)

## Antifibrotic Macrophages: How to Identify and Induce or Recruit Them?

Within fibrotic parts of tissues, higher numbers of macrophages were shown to be present as compared to the healthy parts, and these were shown to be important for fibrosis resolution (61, 67, 68). One of the main tasks of these antifibrotic macrophages is clearance of fibrotic ECM, in particular of fibrillar types of collagen. Macrophages are important sources of various matrix-degrading enzymes, and they can take up partially degraded collagen fragments (6). The expression of these matrix-degrading enzymes and of the receptors for uptake of collagen fragments could therefore potentially be markers of antifibrotic macrophages *in vivo*.

Collagen fibers are cleaved extracellularly by proteases, such as MMPs and cathepsins. Intact fibrillar collagen can only be cleaved by a subset of MMPs (MMP1, MMP8, MMP13, and MMP14) and by other proteases, such as cathepsin K (69–71). Subsequently, collagen pieces are further degraded by other members of the MMP family like MMP2 and MMP9 (6). The main cellular source of matrix-degrading enzymes is macrophages. Huang et al. showed expression of different MMPs in the various macrophage phenotypes *in vitro* (72). Therefore, MMP expression by macrophages might serve as a functional marker to identify antifibrotic macrophages *in vivo*. Scar-associated macrophages were shown to be a source of MMP13 and a strong correlation between the presence of MMP13-positive macrophages, and enhanced regression was shown in fibrotic carbon tetrachloride mouse livers (68). Not only MMP13 but also other members of the MMP family (MMP3, MMP8, MMP9, MMP12, and MMP14) were identified in scar-associated macrophages and associated with resolution activities in liver (73, 74). The presence of MMP-expressing macrophages in scar tissue was also seen in other fibrotic tissues, such as lung, kidneys, heart, and spinal cord. Shechter et al. showed MMP13-expressing macrophages in glial scar tissue and related this to a resolving macrophage phenotype (58). Cabrera et al. showed increased MMP9 expression in alveolar macrophages that appear in the regression phase of the bleomycin-induced lung fibrosis (75). Also, Popov et al. showed that MMP9, in contrast to MMP12 and MMP13, was particularly induced during resolution and higher expressed than during fibrogenesis (74). Within lung and liver, MMP9 expression is particularly observed in macrophages, as can be checked in immunohistochemical stainings provided by the human protein atlas (76).

In addition to MMPs, macrophages also express other ECM-degrading enzymes, such as the cysteine proteases, i.e., cathepsins (71). MMPs are traditionally considered to be the main agents of ECM degradation, but the lysosomal cathepsins can also be secreted into the extracellular space where they can remain proteolyticaly active and degrade various components of the ECM (71). Cathepsin K is the only protease with the ability to degrade intact fibrillar collagen, both at the ends of the fibril and at multiple sites within the triple helix. Overexpression of cathepsin K-protected animals from developing bleomycine or silica-induced pulmonary fibrosis, while deleting it accelerated the development of fibrosis (66, 77, 78). These findings all suggest high antifibrotic activity of cathepsin K, and therefore of macrophages in the lung. Alveolar macrophages in the resolution phase are also reported to produce plasmin, a protease associated with reducing TGFβ1 levels and thus with reduced stimulation of collagen synthesis (64).

Matrix metalloproteinases can also contribute to other activities, such as cellular migration (79) and activation of cytokines and growth factors (80, 81). The expressions and activities of MMPs are therefore not limited to the resolution phase. Certain subtypes are more enhanced during fibrogenesis as compared to resolution, e.g., MMP2 in liver fibrosis (74). This might hamper the use of certain MMPs as markers for antifibrotic macrophages. Based on the current knowledge about the expression patterns of matrix-degrading enzymes in macrophages in fibrosis and resolution, in particular MMP9, MMP13, and cathepsin K seem suitable markers to discern antifibrotic macrophages *in vivo* from other macrophage phenotypes.

In addition to the matrix-degrading activities of antifibrotic macrophages, candidate markers of antifibrotic macrophages could also be proteins involved in the induction of proteolytic enzymes and proteins involved in clearance of degraded ECM proteins. After extracellular degradation, further processing of collagen fragments occurs intracellularly, predominantly in the lysosomal compartments of the cell. To that end, collagen fragments are internalized via phagocytosis, macropinocytosis, or receptor-mediated endocytosis (6).

Phagocytosis, for instance, is mediated by binding of collagen fragments to cellular membrane integrin α2β1. For receptor-mediated endocytosis binding to transmembrane mannose receptor CD206 or mannose receptor 2 (Mrc2; also called Endo180) is required (6, 82–84). López -Guisa et al. showed upregulation of Mrc2 in a subset of macrophages at sites of renal fibrosis directing the process of repair. Renal fibrosis was significantly worse in Mrc2-deficient mice, which was related to lower collagen turnover. In addition, treatment of wild-type mice with a cathepsin inhibitor, which blocks the proteases implicated in Mrc2-mediated collagen degradation, worsened UUO-induced renal fibrosis (83).

The extracellular bridging glycoprotein Mfge8 (milk fat globule-EGF factor 8) has also been described to be involved in the cellular uptake of collagen fragments (6, 65, 85). Atabai et al. showed that Mfge8 decreased the severity of tissue fibrosis in a mouse model of pulmonary fibrosis by binding and targeting collagen for cellular uptake through its discoidin domains (85). Reddy et al. showed that nitrated fatty acids regulated the expression of Mfge8 in alveolar macrophages and thus stimulated collagen uptake and its further degradation (65). The usefulness of these receptors, involved in the cellular uptake of collagen, in identifying antifibrotic macrophages has not been investigated in great detail and will require more studies.

Other proteins expressed by macrophages that have been shown to contribute to the antifibrotic phenotype of macrophages are Arg-1 (17) and FIZZ1 (22). Both were shown to limit Th2-dependent responses that are required for the development of fibrosis.

As is clear from the previous sections, production of matrix-degrading enzymes is one of the key characteristics of antifibrotic macrophages. Therefore, to induce this type of macrophage, it will be helpful to understand the signals involved in attracting these macrophages to the fibrotic areas and/or the signals that induce the expression of matrix-degrading enzymes and collagen uptake receptors. These could be cytokines such as TNFα, IL-1β, IFNα/β, and IL-4, growth factors, chemokines, or even processes (58, 81, 86–88).

Popov et al. showed that the enhanced proteolytic activity of macrophages was induced after phagocytosis of apoptotic cholangiocytes that were increasingly present in the resolution phase of biliary fibrosis (74). The receptor involved in this phagocytosis-induced proteolytic activity was most probably the tyrosine-protein kinase Mer receptor (MERTK), which is highly expressed on macrophages (74, 89, 90). Gene variants of MERTK have been shown to be risk factors for the progression of hepatitis C-induced liver fibrosis (91, 92). Through no functional data of the gene variants of MERTK were shown, making it hard to interpret this data. Similar phagocytosis-induced proteolytic activity was reported in the lung, in which apoptotic cell instillation induced peroxisome proliferator-activated receptor-γ (PPARγ) expression in macrophages and subsequently stimulated resolution of bleomycin-induced fibrosis (93). Whether MERTK and PPARγ are useful markers for antifibrotic macrophages needs to be investigated in further detail. PPARγ seems to be a promising candidate as agonists of PPARγ have been investigated as a possible antifibrotic therapy in multiple settings (65, 94–100).

Some of the cytokines or their receptors that induce antifibrotic behavior are expressed by macrophages themselves; therefore these cytokines or their receptors could potentially also be markers of antifibrotic macrophages. However, their ubiquitous expression by various other cells may hamper their use *in vivo*.

Tumor necrosis factor alpha receptor (TNFαR) or the production of TNFα may be potential inducers and/or markers of antifibrotic macrophages, though this depends on the stage of the disease limiting their use. Macrophages are important producers of TNFα and thereby contribute to inflammation after injury. Inhibiting TNFα at this point has been shown to lead to less fibrosis in models of kidney, liver, heart, and lung fibrosis (101–105). However, TNFα has also been shown to have antifibrotic activities, especially in the resolution stage of fibrosis. Recent research showed that intratracheal delivery of TNFα reduced lung collagen levels and improved lung architecture. In addition, mice deficient in TNFα exhibited delayed resolution of bleomycin-induced pulmonary fibrosis, further showing that TNFα may be important in the resolution phase of fibrosis by inducing antifibrotic macrophages (106). A study in patients with pulmonary fibrosis showed that release of TNFα by macrophages and monocytes of these patients was higher than of controls, which may be a sign that the lung is trying to degrade excess collagen or a sign that inflammation is still important in patients diagnosed with pulmonary fibrosis (107). The fact that anti-inflammatory drugs such as corticosteroids are harmful to pulmonary fibrosis patients indicates that TNFα is probably involved in attempted resolution (108). Production of TNFα by antifibrotic macrophages may have an effect on macrophages themselves through TNFα type 1 and/or 2 receptors or affect other cells. Both in the heart and in the kidney, TNFα type 2 receptor expression on macrophages was found to essential for accelerating fibrosis resolution (109, 110). A recent publication by Lemos et al. showed that the effect of TNFα in muscle fibrosis was through induction of apoptosis of myofibroblast progenitors (111).

Treatment of liver macrophages with interferon-a2b induced a higher MMP13 expression, and these macrophages also showed a higher expression of IL-10 (88). Similar findings were reported in glial scars by Shechter et al. (58). The effect of IL-10 on fibrosis, however, is not clear since increased levels of IL-10 were accompanied by reduced fibrosis in one study (73), while other studies have reported that IL-10 acts profibrotic (112, 113).

Cytokines and chemokines that are involved in recruitment antifibrotic macrophages are macrophage migration inhibitory factor (MIF), CX3C ligand 1 (fractalkine), and vascular endothelial growth factor (VEGF) (60, 61, 114). CD74, CXCR2, and CXCR4 are receptors for MIF, and their expressions appear to be associated with recruitment of resolving macrophages (61, 115, 116). This also is the case for chemokine receptor CX3CR1, and this receptor may also be helpful in the detection of antifibrotic macrophages (60). Another chemokine involved in the recruitment of resolution-promoting monocytes appears to be VEGF. Treatment with a neutralizing antibody against VEGF during fibrosis resolution delayed resolution, and this was shown to be dependent on CXCL9 and MMP13 (114). In addition, enhanced expression of CXCL10 in macrophages has been shown to accelerate resolution of pulmonary fibrosis (59, 117, 118). Interestingly, in the study by Tighe et al., IFNγ was found to be able to stimulate production of CXCL10 in macrophages, and this may therefore contribute to the known antifibrotic effects of IFNγ (59, 119).

In conclusion, various studies indicate the existence of antifibrotic macrophages that play a key role in resolving fibrotic ECM, and therefore these macrophages may be a target for therapeutic intervention. Identification of this subset *in vivo* is not easy, but various options can be explored. One of the most obvious is the expression of matrix-degrading enzymes in macrophages, in particular, MMP9, MMP13, and cathepsin K. Other options include the chemokines CXCL10 and CXCL9, chemokine receptor CX3CR1, M2 markers Arg-1, FIZZ1, and PPARγ, collagen-uptake receptors MRC1, MRC2, and MFGE8, and cytokines like TNFα (see also Table 3). However, most of these proteins are not specific to macrophages and even the different phenotypes of macrophages in the proinflammatory/fibrotic phase and in the resolution phase seem to use them.

**Table 3:** **Markers of antifibrotic macrophages and potential therapeutic approaches inducing or attracting antifibrotic macrophages or inhibiting the recruitment of profibrotic monocytes**.

Markers	Prospective drug
TNF receptor (109, 110)	TNFα (106)
CX3CR1 (60)	RANKL (122–124)
TNFα (106, 107, 111)	PPARγ agonist (93–100)
CXCL10 (59, 117, 118)	IFNγ (59, 125)
CXCL9 (117, 118)	IFNα (58, 88)
MMP9 (73–75)	Asprin-triggered lipoxin A analogs (126)
MMP13 (68, 118)	CCL2 inhibitors (62)
Cathepsin K (66, 71, 77, 78)	
MERTK(89, 90)	
PPARγ (93–100)	
MRC1 (120)	
MRC2 (121)	
MFGE8 (6, 65, 85)	
Arg-1 (17)	
FIZZ1 (22)	

Induction or recruitment of antifibrotic macrophages is even less well defined. Monocytes that turn into antifibrotic macrophages appear to be recruited by CX3C ligand 1 or VEGF. Cytokines that can induce antifibrotic behavior of macrophages in well-defined circumstances are TNFα, IFNα, or IFNγ.

## From Concept to Market: Therapeutic Applications and Challenges

As antifibrotic macrophages can be crucial in the resolution of fibrosis in various organs, they constitute a valid novel target for therapeutic intervention. Therefore, understanding of how to specifically induce their beneficial activities may lead to a generation of new antifibrotic compounds.

In addition to the aforementioned TNFα, IFNγ, and IFNα, only a few potential therapeutic compounds affecting antifibrotic macrophages have been described in literature. One of the few examples is the use of PPARγ agonists that can induce antifibrotic properties in macrophages. Experimental studies in kidney, liver, heart, and lung have shown that various PPARγ agonists can alleviate fibrosis, though not all have investigated macrophages specifically (65, 94–100). There is even phase 1 safety study in clinicaltrials.gov describing the use of PPARγ-agonist rosiglitazone for the treatment of focal glomerulosclerosis. This study ended in 2007, but no results have been posted yet.

A currently unexplored option is the possible use of receptor activator of nuclear factor-κB ligand (RANKL). Many tissue macrophages express the receptor RANK for this ligand, and there are several studies showing that RANKL stimulation induces the release of proteases, which can degrade ECM (76). Wittrant et al. showed that RANKL stimulated MMP9 and cathepsin K expression (122), and Matsumoto et al. also showed that RANKL induced cathepsin K gene expression (123). Another study showed that RANKL, through binding to RANK, activated the nuclear factor-κB pathway and induced MMP9 expression. They also suggested that by costimulating with IL-1β or TNFα, it was possible to synergize with RANKL to further enhance MMP9 expression (124). We are currently investigating whether RANKL can indeed induce antifibrotic macrophages in settings of established fibrosis.

**Figure 1:**
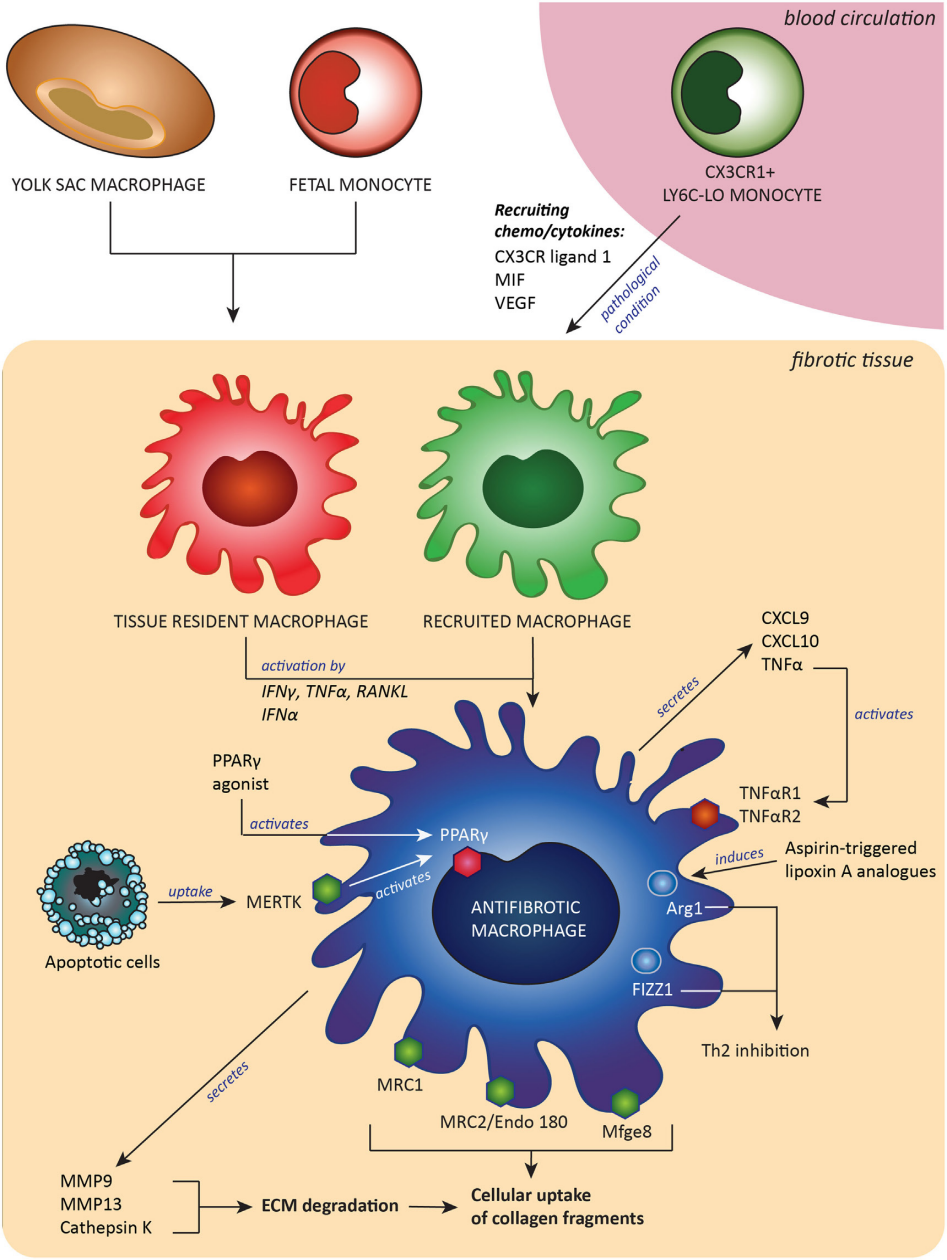
**Antifibrotic macrophages, derived from either embryonic tissue macrophages and/or Ly6C-lo monocytes, contribute to fibrosis resolution by expressing extracellular matrix (ECM)-degrading enzymes and receptors to take up pieces of degraded ECM and by expression of proteins that downregulate Th2-associated inflammation**. These antifibrotic macrophages can be induced or attracted by a number of signals, such as cytokines, chemokines and growth factors. Abbreviations: MIF, macrophages migration inhibitory factor; CX3CR ligand 1, ligand for C–X3–C motif chemokine receptor 1; VEGF, vascular endothelial growth factor; CXCL9 and -10, C–X–C motif chemokine ligand-9 and -10; RANKL, receptor activator of nuclear factor-κB ligand; TNFα, tumor necrosis factor α; TNFαR1/2, tumor necrosis factor receptor type 1 or 2; IFNγ, interferon γ; IFNα, interferon α; MMP9 and MMP13, matrix metalloproteinases 9 and 13; Mfge8, milk fat globule-EGF factor 8; MERTK, tyrosine-protein kinase Mer receptor; Mrc1 and Mrc2, mannose receptors 1 and 2; PPARγ, peroxisome proliferator-activated receptor-γ; Arg-1, arginase-1; FIZZ1, resistin-like molecule alpha 1; Th2, T helper 2 lymphocyted-mediated.

Another option described was the use of a Spiegelmer-based inhibitor of CCL2, named mNOX-E36, that was found to inhibit recruitment of Ly6C-hi monocytes and thereby accelerated resolution of liver fibrosis (62). The last of the few examples was a synthetic analog of asprin-triggered lipoxin A(4). Lipoxins have potent proresolution effects, and this synthetic analog called ATLa reversed collagen deposition by inducing Arg-1-positive macrophages in a bleomycin model of pulmonary fibrosis (126). A summary of the origin and all characteristics of antifibrotic macrophages is depicted in Figure 1.

One factor worth considering is the translation of these results in rodents to the human situation. As said before, an obstacle in this translation is that most knowledge so far is obtained with mouse models, and the markers and effector molecules of antifibrotic macrophages in humans are largely unexplored (127).

In addition, several fibrosis-inducing agents, such as carbon tetrachloride, bleomycine, silica, and nutritional interventions, are highly effective in establishing advanced fibrosis in mice, but they do not represent key elements of human disease completely.

## Conclusion

The flurry in new studies investigating antifibrotic behavior of macrophages in recent years has made the elusive antifibrotic macrophage slightly more tangible. This subset of macrophages appears to be derived from embryonic tissue-resident macrophage or recruited Ly6C-lo monocytes and expresses a variety of markers traditionally assigned to both M1 and M2 macrophages, including MMP9, MMP13, cathepsin K, CXCL10, CXCL9, CX3CR1, Arg-1, FIZZ1, PPARγ, MRC1, MRC2, MFGE8, and TNFα. Although therapy aimed at the antifibrotic macrophage is still in its infancy, it is expected that more targets for therapeutic entities will appear when antifibrotic macrophages are better understood.

## Author Contributions

AA and KP collected data, summarized findings, wrote the manuscript, and designed the figure; LB interpreted findings described in literature, rewrote parts of the text, provided critical comments, and reviewed and approved the manuscript; and BM designed the outline of the review, interpreted findings described in literature, rewrote parts of the text, provided critical comments, and reviewed and approved the manuscript.

## Conflict of Interest Statement

The authors declare that this review was written in the absence of any commercial or financial relationships that could be construed as a potential conflict of interest.
